# The web of intrigue: unraveling the role of NETosis within the gut-microbiome-immune-heart axis in acute myocardial infarction and heart failure

**DOI:** 10.1097/XCE.0000000000000309

**Published:** 2024-08-08

**Authors:** Tai Yasuda, Kate Deans, Aditi Shankar, Robert Chilton

**Affiliations:** a Department of Anesthesiology, University Hospital, UTHSC San Antonio; b Department of Cardiology, South Texas Department of Veteran Affairs; c Department of Cardiology, University Hospital, UTHSC San Antonio, San Antonio, Texas, USA

**Keywords:** acute myocardial infarction, heart failure, microbiome, NETosis

## Abstract

This review summarizes the role of NETosis, or the release of neutrophil extracellular traps (NETs), and its interplay with the gut microbiome in acute myocardial infarction (AMI) and heart failure. NETosis contributes to inflammation, thrombosis, and atherothrombosis, all central to the pathophysiology of AMI and heart failure. NETosis can be activated by inflammation and dietary factors, indicating association with metabolic conditions. In cases of heart failure, NETosis is regulated by inflammatory molecules such as C-reactive protein (CRP), and Krüppel-like factor 2 (KLF2) – a protein that plays a role in controlling inflammation, and angiotensin II. Changes in the gut microbiome are linked to the severity and recovery of cardiac injury post-AMI and heart failure progression. The microbiome’s influence extends to immune modulation and inflammatory responses, potentially affecting NETosis.

## Acute myocardial infarction and NETosis

NETosis, involving the release of neutrophil extracellular traps (NETs), plays an important role in acute myocardial infarction (AMI) [1]. In this process, neutrophils expel a meshwork of DNA fibers embedded with histones and enzymes like myeloperoxidase (MPO), which trap and neutralize pathogens but also contribute to inflammation and thrombosis [2]. This can be particularly relevant in the context of AMI, where inflammation and thrombosis are key pathological features [1].

During AMI, the formation of NETs has been linked to atherothrombosis. Atherosclerotic lesions can lead to plaque rupture, exposing thrombogenic material to blood cells and triggering platelet and leukocyte recruitment. Activated neutrophils, in response to this environment, release NETs, which further enhance platelet activation and aggregation [3]. This platelet–neutrophil interplay is a critical factor in the development and stability of arterial thrombosis, a central event in AMI [4].

Furthermore, NETs contain various prothrombotic and proinflammatory molecules, which exacerbate the thrombosis process and can lead to additional endothelial damage or dysfunction [5]. In this way, NETosis is closely intertwined with the pathophysiology of AMI, contributing to both the formation of thrombi and the progression of heart damage [6].

Interestingly, studies have also explored the relationship between glucose levels and NETosis in patients with AMI [7], suggesting a potential interaction between metabolic status and the severity or characteristics of AMI [8]. Elevated glucose levels might influence the formation or activity of NETs, although this relationship requires further clarification [9].

Overall, understanding the role of NETosis in AMI provides valuable insights into the disease’s pathophysiology and highlights potential therapeutic targets to mitigate the inflammatory and thrombotic components of heart attacks [10,11].

## Heart failure and NETosis

NETosis plays a notable role in the pathophysiology of heart failure [12]. Neutrophils, which are key players in this process, extend beyond their traditional antimicrobial functions to participate in sterile inflammation and disease progression. In the context of heart failure, particularly with conditions like cardiac hypertrophy, the formation of NETs can contribute to the disease’s development and severity [13].

In studies involving heart failure, especially those induced by angiotensin II in animal models, it has been demonstrated that neutrophil depletion can reduce cardiac hypertrophy and dysfunction [14]. This indicates a clear involvement of neutrophils and NETosis in the progression of heart failure [15]. Additionally, the involvement of Krüppel-like factor 2 (KLF2) in neutrophils has been shown to oppose NET formation, suggesting that targeting this pathway could be a potential therapeutic strategy.

Furthermore, the presence of C-reactive protein (CRP) in the serum has been linked to the induction of NETosis [11]. Patients with heart failure and higher levels of CRP have demonstrated an increased formation of NETs, which in turn can lead to aggravated vasculature injury and associated cardiovascular events. This connection between CRP levels, NETosis, and heart failure provides insights into the underlying mechanisms of the disease and highlights potential therapeutic targets [16].

In summary, the interaction between neutrophils, NET formation, and factors such as KLF2 and CRP is crucial in the progression and exacerbation of heart failure [13]. Understanding these relationships opens avenues for novel therapeutic interventions targeting neutrophil function and NETosis in heart failure management.

## Microbiome and acute myocardial infarction

The microbiome, particularly the gut microbiome, appears to impact the incidence and progression of AMI. Research has shown that changes in the gut microbiome can influence the severity of cardiac injury and recovery post-AMI [17].

One study found that the gut microbiome composition and its diversity were predictive of the response to ischemia/reperfusion (I/R) injury in myocardial infarction [18]. This suggests that the gut–microbiome–immune–heart axis plays a crucial role in the pathology of AMI, offering new insights for potential gut-targeted therapeutic strategies to improve AMI prognosis.

Additionally, gut microbial translocation, where bacteria or bacterial products move from the gut to the bloodstream, has been associated with inflammation and cardiovascular events after ST-elevation myocardial infarction (STEMI). Specifically, increased levels of microbial translocation markers like lipopolysaccharide (LPS) and d-lactate were observed post-STEMI, which correlated with inflammatory responses and changes in left ventricular function. This highlights the interplay between the gut microbiome, systemic inflammation, and heart function in the context of myocardial infarction [19].

Overall, these findings emphasize the importance of the gut microbiome in cardiovascular health and disease, particularly in the context of AMI. The gut microbiota can influence heart function, offering a novel perspective on the management and treatment of myocardial infarction.

## Microbiome and heart failure

The gut microbiome plays an important role in the development and progression of heart failure [20]. It has been linked to various aspects of heart failure pathophysiology, largely through its impact on chronic inflammation and immune modulation [21]. A notable factor in this relationship is gut dysbiosis, which is characterized by an imbalance in the microbial composition of the gut. This dysbiosis can lead to an increase in pathogenic bacteria and a decrease in bacteria that produce beneficial short-chain fatty acids (SCFAs) [21].

Moreover, an increased intestinal permeability in heart failure patients allows for the translocation of microbial products, such as lipopolysaccharides and peptidoglycans, into the bloodstream [22]. This translocation is associated with the progression of heart failure, as these microbial products can trigger systemic inflammation and immune responses that exacerbate cardiac dysfunction [23].

In addition to this, metabolites produced by the gut microbiota, such as trimethylamine N-oxide (TMAO), have been linked to cardiovascular risk [24]. TMAO is produced from dietary elements like choline, lecithin, and L-carnitine, primarily found in animal products, through the action of gut microbiota. Elevated levels of TMAO are associated with an increased risk of cardiovascular diseases, including heart failure [25]. These metabolites affect the body in various ways, including altering cholesterol and bile acid metabolism and activating inflammatory pathways [26].

Therefore, understanding the interactions between the gut microbiome and heart failure is crucial for developing new therapeutic strategies [27]. Modulating the gut microbiota and targeting specific microbial metabolites represent potential avenues for treatment. This approach could lead to individualized treatments for heart failure patients based on the composition and function of their gut microbiome.

## Microbiome and NETosis

The microbiome, particularly the gut microbiome, has a substantial influence on various aspects of human health, including immune responses and, potentially, NETosis [28]. The gut microbiome’s interaction with the immune system is complex and multifaceted, affecting both innate and adaptive immunity [29].

Diet is a major factor influencing the microbiome. For example, different types of dietary proteins can alter the composition of the gut microbiota, impacting overall microbial diversity. Proteins from various sources, like animal-based protein, whey, or vegetarian sources like pea protein, have differing effects on gut microbiota. Animal-based proteins tend to increase counts of bile-tolerant anaerobes such as Bacteroides, Alistipes, and Bilophila. In contrast, whey and pea proteins can increase beneficial bacteria like Bifidobacterium and Lactobacillus. These changes in microbiota have implications for health, including inflammation and disease states like inflammatory bowel disease (IBD) and cardiovascular disease [30].

Additionally, dietary fats also influence the gut microbiota. Different types of fats, such as saturated, trans, mono, and polyunsaturated fats, have varying impacts on the microbiota composition and, subsequently, on host metabolism and immune responses. High-fat diets, particularly those rich in saturated fats, can lead to changes in the microbiome that promote inflammation and metabolic disorders.

Moreover, the gut microbiota can modulate responses to certain therapies, such as immunotherapy, by influencing immune cells like dendritic cells, monocytes/macrophages, and natural killer (NK) cells. This interaction suggests a leading role of the microbiota in shaping systemic immune responses, which could include processes like NETosis [31].

The direct relationship between the microbiome and NETosis, however, particularly in specific conditions like infections or cardiovascular diseases, is still an area requiring more research [32]. Understanding this relationship could open new avenues for therapeutic interventions targeting the microbiome to modulate NETosis-related pathologies [33].

## Summary

**Fig. 1:**
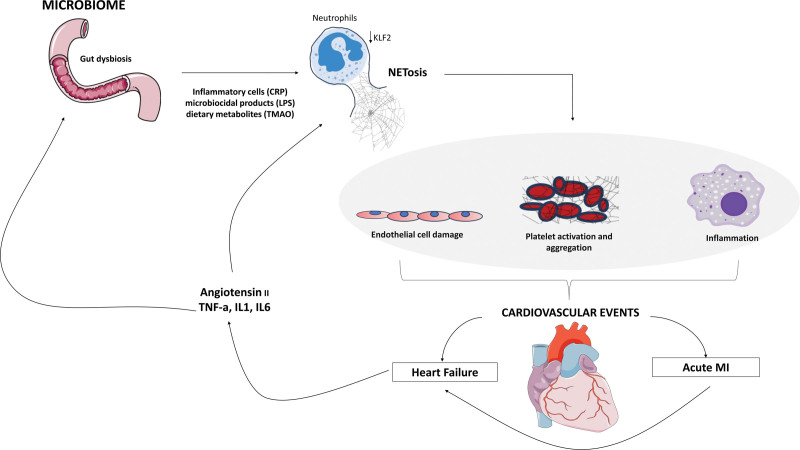
Proposed schematic representation of the gut-microbiome-immune-heart axis. Gut dysbiosis, characterized by an imbalance of the microbial composition of the gut, is influenced by various factors related to cardiometabolic disease. Inflammatory processes, for example, release inflammatory molecules like CRP and increases gut permeability, allowing for translocation of microbial products such as LPS. High fat diets alter gut microbiome by increasing gut permeability and releasing metabolites such as TMAO, which directly activates inflammatory pathways and is associated with cardiovascular disease. In response to these inflammatory molecules, NETs are released by activated neutrophils, specifically by the enhanced proinflammatory function of KLF2 deficient neutrophils. NETs are comprised of a complex web of DNA fibers, antimicrobial histones, and enzymes such as MPO. These components are directly cytotoxic, prothrombotic, and further activate inflammatory pathways. Thus, NETs promote endothelial damage, thrombosis, and inflammation, all central to the pathophysiology of cardiovascular events such as acute myocardial infarction and heart failure. In turn, heightened renin-angiotensin activity in heart failure leads to hyper physiological levels of AngII which has been postulated to mediate the KLF2/NETosis/thrombosis pathway leading to small vessel dysfunction. Additionally, proinflammatory cytokines such as TNF-a, IL1, and IL6 are released in these disease states which leads to increased gut permeability and NETosis, further activating this vicious cycle. CRP, C-reactive protein; KLF2, Krüppel-like factor 2; LPS, lipopolysaccharides; MPO, myeloperoxidase; NETs, neutrophil extracellular traps; TMAO, trimethylamine N-oxide.

NETosis, a critical process involving the release of NETs, impacts AMI and heart failure (Fig. 1). This complex biological response is due to neutrophils discharging a DNA mesh infused with enzymes and histones, designed to trap pathogens but also inadvertently contributing to inflammation and thrombosis, central to AMI’s pathology. The formation of NETs is intrinsically linked to atherothrombosis, particularly notable during the rupture of atherosclerotic plaques. This event further activates neutrophils, leading to enhanced platelet aggregation and increased arterial thrombosis risk, a hallmark of AMI. Additionally, NETs comprise various molecules that intensify thrombosis and cause further endothelial damage. Intriguingly, emerging research indicates a possible link between NETosis and metabolic states in AMI patients, such as elevated glucose levels, hinting at broader implications of this process [34]. The study of NETosis in the context of AMI and heart failure is pivotal, offering insights into disease mechanisms and highlighting new avenues for therapeutic interventions to alleviate inflammation and thrombotic complications associated with heart disease.

## Acknowledgements

### Conflicts of interest

There are no conflicts of interest.
